# Prospectus of a Medical Bill for Norway. Presented by the Royal Commission

**Published:** 1845-01

**Authors:** 


### Art. XVIII.

*Udkast til Lov om Medicinalvæsenet i Norge, &c.—Christiania*, 1844.
*Prospectus of a Medical Bill for Norway. Presented by the Royal Commission.—Christiania*, 1844. 8vo, pp. 188. (*June* 14, 1844.)

While in Great Britain we are at the present moment zealously labouring for the amendment of the legislative acts relating to the practice of the healing art, it is gratifying to observe, that in other lands a similar necessity for medical reform is called for by the altered position and by the improved education of medical men.

It is generally urged, by those opposed to any change of the present system in this island, that the physician, surgeon, and apothecary, in foreign countries, are the mere tools of a despotic government, and that in Great Britain alone, true liberty is enjoyed by the medical practitioner. Whether such liberty is preferable to the protection accorded to the profession in Austria or in Prussia, is a question we will discuss elsewhere; but we may adduce the present outline of a medical bill for Norway, as a proof that it is not *despotic* governments alone which have found it necessary to revise and improve the laws which regulate medical practice. By a singular coincidence, medical bills for England and for Norway have appeared within two months of each other, and each has been promulgated by the ministers of the crown under very similar circumstances. Sir James Graham has left his bill for the space of six or eight months to the consideration of the profession, while Dr. Holst and his colleague have at the command of the Government Education Commission, drawn up and published the present bill for Norway, to be submitted to a similar ordeal before it is presented to the Storthing or parliament of that country. From the preface we learn that the commission have published the present outline of a bill, to obtain from all those whose knowledge of the subject entitles them to consideration, the most complete expression of their opinions as to its merits or defects.

The bill is divided into seventeen chapters, which occupy the first sixty pages; the remaining 120 pages being devoted to long notes and explanations of its various clauses. In these notes or appendix, we find the fullest account of the reasons which have influenced the framers of the bill in forming its various enactments; so that not only the bare measure itself, but the very motives which have guided its authors, are frankly submitted to the consideration of the medical profession.

Clause i forms the very groundwork of the whole bill, by creating a medical college or council for the exclusive superintendence of all affairs relating to the medical profession.

The old Norwegian College of Health, which held its sittings in Copenhagen, during the union of Denmark and Norway, was, of course, dissolved when the latter kingdom was united to Sweden; and up to the present day its medical regulations were directed by no less than three different departments of the government; viz., that of the church, of the army, and of the navy. The inefficiency of such a divided administration had long been deeply felt, and consequently the first effect of the new bill will be, to establish in Norway, as is proposed also in England, a central council of health and medical legislation.

The council will consist of only three members, a number which is quite sufficient to regulate the medical affairs of so small and thinly-peopled a kingdom as Norway. The members will be nominated by the government; two being chosen from among physicians in the civil department, and the third from those attached to the army. A fourth, or extraordinary member, is to be chosen from among the apothecaries; but his assistance will only be required in cases relating to chemistry and to pharmacy.

The College will exercise a careful superintendence of all practitioners in medicine, and will publish an annual list of those who are qualified to practise in every branch of the healing art.

Clause ii treats of the duties of medical men in general. No one is to be allowed to practise medicine in Norway, who has not passed his examination at the University of Christiania. All who practise withont being duly licensed will be punished by fines and imprisonment. The king and his royal court are, however, allowed to employ foreign physicians, though it does not seem to be inferred that they may intrust themselves to the power of quacks or unqualified individuals.

It appears that in Norway as in most continental states, the country is divided into official districts, to each of which there is appointed a medical officer. This does not however, exclude others from settling in the same districts; but the above practitioner becomes a sort of government official, whose duty it is to report immediately the outbreak of any epidemic, either among the human race, or cattle, to the superintending physician, or inspector, who has several districts under his care.

No medical practitioner may trade in drugs, or have any connexion with an apothecary's shop. (But in remote country districts he may keep medicines for his patient's use, if no apothecary's shop be within reach.)

A medical man who shall exhibit gross ignorance in the practice of his profession may be struck off the roll of qualified persons, but he shall not be amenable to punishment by common law.*

Clause iii. Of district inspectors. (Amts Læger.) Each inspector shall have several districts under his command, and shall be at the head of the practitioners within his department. He himself, however, is in some measure subject to the lay officers of the government, as he cannot leave his department for twenty-four hours without permission from the presiding magistrate. He will act at the same time as a kind of registrar-general, and will receive, every January, the reports from the several district practitioners as to their sanitary condition.

When a practitioner settles in any district he is bound immediately to give notice thereof to the district inspector.

Clause iv. Of district practitioners. They are, as before noticed, subject to the inspector-physician of districts, and are obliged to give notice immediately of any infringement of the regulations regarding practice. All these officers receive a salary from the government, and upon their death a pension is settled on their widows. They are obliged every year to furnish a report of the sanitary condition of their district.

Clause v. Of inspector-physicians of towns. (Stadts Læger.) One is

* We may here observe that the science of medicine and surgery are comprehended in Norway under one head, and that although the words physician and surgeon are occasionally employed, the name of "Læge" or "healer" is that which in the Danish language expresses every practitioner of the healing art. Perhaps the popular meaning attached to the name of *Doctor* in this country approaches nearest to the Danish appellation of Læge. The licentiate in medicine and surgery of Sir James Graham's Bill would come nearer to the "Læge" of Norway.

appointed to each of the three capital cities of Norway, and also to such other of the large towns as the government shall decide them to be necessary for. They rank with the country inspectors of districts, and their duties are of a similar description.

Clause vi. Of midwives. For these, too, Norway is divided into districts, but the number of midwives for each is to be determined by the local magistracy. They are required to undergo a strictly defined course of study, the expense of which, if they themselves cannot defray it, is to be borne by the commune to which they belong; and should they subsequently wish to relinquish their profession, they must repay to the commune the sum expended upon their education. A certain pension is allotted to those who become superannuated; and this usually is about two thirds of their annual income. Every female, however, is at liberty to employ, if she think fit, an uneducated or unlicensed female as midwife, but the individual so employed may not interfere *medically* with the labour, and if she be proved to have done so, she shall be liable to a fine of from two to five dollars. This is surely an unnecessary provision, as it opens the door to much unqualified practice; and it would, we think, be very difficult to define the extent of assistance which may be given without incurring a penalty.

Clause vii relates to infections and epidemic diseases. Immediate notice is to be given of the outbreak of them by the district practitioners.

Clause viii. Of vaccination. No individual can enter as a scholar in any school or institute, private or public, nor may he be confirmed or married, without affording satisfactory proof of having had either cowpox or smallpox. But this regulation does not apply in cases where it shall be proved that the parties have been vaccinated twice without effect. In case of smallpox breaking out in any house or village in the country, each inhabitant shall be bound to have himself vaccinated, unless he have previously had smallpox, or have been vaccinated *successfully* within the last five years. Inoculation for smallpox is declared penal, and punishable by fine and imprisonment.

Clause ix. Of quarantine regulations. This is a chapter of great length, and enters fully into the various regulations for the protection of Norway from infectious diseases brought by shipping. A distinction is made between the period of quarantine for yellow fever or for cholera, and that for the oriental plague. Where the plague actually exists on board, no ship can be freed within less than thirty days, but if the vessel contain goods liable to transport the infection, and yet no case of plague be proved to be on board, or to have occurred during the passage, the term of detention is only twenty days. If the vessel come from a suspected port, ten days are deemed sufficient.

Clause x regulates provincial hospitals and infirmaries.

Clause xi regards the army medical staff.

Clause xii. Of dentists. They are not allowed to exercise more than the simple art of drawing, filing, and replacing teeth; and all surgical operations on the jaws are strictly forbidden, unless they be at the same time authorized medical practitioners.

Clause xiii. Of veterinary surgeons. None are permitted to practise without a regular education and license.

Clause xiv. Of apothecaries (or chemists and druggists.) In all countries excepting in England, the trade of the apothecary is strictly separated from the profession of physic. InNorway, as in other continental states, the apothecary is a well-educated individual, who must obtain a regular license, after undergoing a strict course of instruction in chemistry, pharmacy, and the collateral sciences. With a population rather less numerous than that of London, Norway has only forty-five licensed apothecaries, but it must be remembered that in all remote districts the medical practitioner is allowed to keep a certain amount of drugs, for which, however, he is only permitted to charge his patients 10 per cent. above the trade price at which he receives them from the apothecaries.

The course of preparatory study for an apothecary's license is, in our opinion, not sufficiently extended. After serving for three years as an apprentice in an apothecary's shop, he is to be examined by the deputy inspector-physician of the district, as to his possessing the amount of knowledge necesssry for the more responsible situation of assistant. Should he be found sufficiently qualified, he must remain at least *two years* in this second capacity, ere he can be admitted to his pharmaceutical examination ; and during this period, his master is bound to afford him every means of acquiring the necessary knowledge. Having passed his pharmaceutical examination, he then receives his license as an apothecary, but must wait till a vacancy occurs, to commence business on his own account.

A fine of ten specie dollars is imposed upon every apothecary who shall sell any medicines without a prescription from an authorized practitioner. But certain of the simpler drugs and preparations may be sold "over the counter ;" the number and nature of these being determined by the College. The price of drugs is fixed annually by a published tariff; and whoever demands more than legal charge for medicines is fined.

No poisons can be sold without a physician's order ; and if any be required for mechanical, chemical, or manufacturing purposes, they may not be delivered to the parties demanding them, unless an attestation be produced, signed by the clergyman or bailiff, stating for what purposes they are required. All poisons are to be delivered sealed up in a black paper, with the word *poison*, and its nature, inscribed thereon.

Clause xv. Of interments of corpses, &c. All burial-grounds that may be formed for the future, must be placed at least 1000 ells from the nearest houses of the town or city; and no grave either there or in the old cemeteries within the town, can be reopened until the lapse of twenty years from the date of the last interment.. No bodies shall be interred in any vault beneath any church or chapel in the kingdom (whatever prescriptive right thereto may be claimed), unless the corpse be hermetically sealed, in a chest of metal, or unless it be certified by a legal practitioner that it has been properly and completely embalmed. The corpses of those who have died of pestilential diseases are to be buried within forty-eight hours after death ; and in such cases, no assemblage of friends to mourn in the chamber of death (sörgestuer), will be permitted ; nor must the body be carried to the grave by bearers, but conducted thither in a hearse. The post-mortem examination of the body may not be refused by the friends, if demanded by the physician who has been in attendance on the case. The bodies of criminals and those who die under sentence of perpetual imprisonment, are to be given to the Anatomical School, for dissection; as also the corpses of those who die in the hospitals, unless their relatives shall object to their being thus disposed of.

Clause xvi. Of remuneration for medical services. All medical practitioners are entitled to recover for attendance; and, in case of the death of the patient, their claims are to be considered before those of the other creditors. A certain tariff of charges is laid down for all occasions, and the fines for exceeding these are exceedingly heavy, amounting to from one to fifty dollars for the first offence, and from ten to two hundred dollars for the second. From the great distance which separates habitations in Norway, much time is lost upon journeys, and consequently a certain mileage is laid down in the tariff; and the practitioner is also entitled to remuneration, if he spend several hours with the sick person.

For each post-mortem examination performed on account of the government, or at the *request* of friends, he is entitled to receive the sum of five specie dollars.

Clause xvii, and last, repeals all former statutes.

We trust that this analysis of the proposed Norwegian bill will not be unacceptable to our readers at the present interesting crisis of British medical affairs. In many respects, the bill is far more extended and comprehensive than the measure of Sir James Graham; but we must remember that in Norway there already existed a connexion between the medical profession and the state, which, up to this period, had never been recognized in Great Britain. Both bills, however, assert, as the basis and groundwork of true medical reform, the necessity of the Council or College of Health and Medical Education, and which shall be in connexion with the government. The ordinances relating to medical police, comprised in the Norwegian bill, are of course not to be found in the proposed measure of Sir James Graham; for in England, with its vast and intricate code of laws, the various questions of medical police would require separate consideration, on account of the vast power of opposing interests—commercial and otherwise—which would have to be conciliated or attacked. There is, however, one portion of the Norwegian bill, which will enlighten many in England on the question of illegal practitioners. None of our readers will fail to notice the decided tone adopted, as regards unqualified practitioners, and the summary punishment to be inflicted on all who transgress in this respect; nor can we discover in the copious explanatory notes of the appendix, that the slightest doubt is expressed by the authors of the bill, as to the reception of this part of their proposed measures. The idea of even indirectly legalizing quackery was reserved for England alone; nor can we wonder, therefore, that while the laws of all other European states are from time to time appealed to, as authorities for some of their proposed enactments, the medical legislature of England is never once hinted at by the learned authors of the Norwegian bill. And this omission is surely not accidental; nor can it arise from ignorance of the English language or laws, with which Dr. Holst is well known to be admirably conversant. We fear that he has found our medical legislature so imperfect and scanty, so inferior to that of other countries, which, in our insular pride, we regard as beneath us in civilization, that he has met with no one enactment, out of the few which we possess, worthy of being translated to his native land.

